# Antioxidant Activity of Polyphenols, from *Mauritia flexuosa* (Aguaje), Based on Controlled Dehydration

**DOI:** 10.3390/molecules27103065

**Published:** 2022-05-10

**Authors:** Hichem Bensaada, María Fernanda Soto-Garcia, Juan Carlos Carmona-Hernandez

**Affiliations:** 1 Medical School, Ferhat Abbas University of Setif 1, Setif 19000, Algeria; dr.bensaada.h@gmx.com; 2 Grupo de Investigación Médica, Línea Metabolismo-Nutrición-Polifenoles, Medical School, Universidad de Manizales, Manizales 17000, Colombia; mfsoto79169@umanizales.edu.co

**Keywords:** polyphenols, antioxidant activity, *Mauritia flexuosa* (aguaje), controlled dehydration

## Abstract

Plant polyphenols offer several benefits for the prevention of diverse illnesses. Fruit’s edible and inedible parts (pulp, seeds, peels, stems, flowers) are important sources of polyphenols. Different industrial processes for fruit treatment and commercialization affect the total polyphenol content (TPC), and probably the biological activity. The purpose of the present work was to determine the TPC and antioxidant activity (by DPPH) of polyphenols extracted from the pulp and seeds of *Mauritia flexuosa* (aguaje), in fresh and dehydrated forms, in order to determine the possible connection with the quantity of polyphenols and their specific antioxidant activity. The highest phenolic content for *M. flexuosa* seeds in fresh form (non-dehydrated) was 270.75 mg GAE/100 g with a 96-h extraction. With respect to the dehydrated samples, the best yield was quantified in the 96-h dehydrated seed sample. For all pulp and seeds, dehydrated for 24, 48, and 96 h, TPC showed a slightly decreasing pattern. The DPPH results were the highest in the 96-h dehydrated samples and the differences among all dehydrated pulp and seed samples were minimal. More studies testing the presence of other antioxidant components could help in understanding the detailed antioxidant activity, and related more to the specific action, rather than only total polyphenol content.

## 1. Introduction

The antioxidant benefit of fruit consumption is mainly connected to the polyphenol content; these types of phytochemicals display several other functions [1,2]. Multiple publications have reported improved lipid metabolism in overweight and obese humans due to a regular diet inclusion of plant polyphenols [3,4,5]. These phytochemicals offer a protective activity, leading to health benefits; several studies relate oxidative stress with the development of diseases such as cardiovascular diseases, neurodegenerative disorders, and cancer [6,7]. With respect to ovarian cancer, there is histological evidence of aortas showing promising anti-proliferative and anti-inflammatory effects and leading to a reduction of cancer cell viability [8,9]. Given the multiple health benefits of polyphenols, it is of general interest to know the phenolic content and major phenolic compounds, such as flavonoids, found in regularly harvested and consumed fruits [10,11,12].

The oxido-reducing activity of compounds containing phenolic rings is the most studied biological property in plant polyphenols. This type of chemical reaction is representative of a misbalance in oxidizing and reducing compounds; which at the cellular level can lead to molecular damage [13]. Plants and fruits can contribute to the reduction of negative side effects in different pathologies.

**Figure 1:**
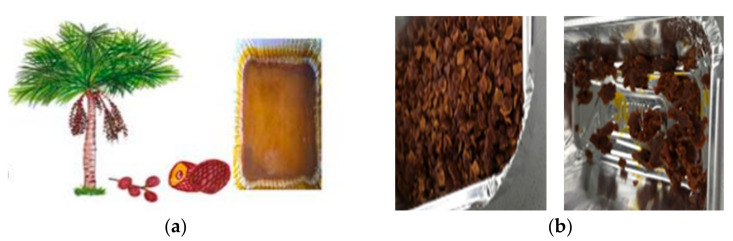
(**a**) *Mauritia flexuosa* (aguaje) palm and fruit (seedless pulp) [2]. (**b**) Dehydrated aguaje samples, seeds and pulp.

Commonly known as buriti plant, *Mauritia flexuosa* (Arecaceae) is a palm broadly cultivated in Colombia, Venezuela, the Guianas, Trinidad, Ecuador, Peru, Brazil, and Bolivia [2,14]. The fresh form of *M. flexuosa* fruits (Figure 1) has an orange, soft, water-soluble, and edible pulp and numerous small circular dark red and brown flat seeds. Several South American aboriginal groups use this Amazonic fruit for medicinal purposes [15,16,17,18,19]. The mesocarp oil is used to treat respiratory symptoms, pneumonia, influenza, snake bites, and heart problems [20]. In Colombia, *M. flexuosa* is also named “aguaje” [2].

Different variables can affect the total polyphenol content (TPC) and the antioxidant capacity of fruits, such as passing from a fresh to a dehydrated long-shelf-life state. This is still an area of active research, while conflicting results have been reported in the literature [21,22,23]. Information is lacking with respect to the specific activity and bio-accessibility of polyphenols, especially due to structural alterations, such as ring modifications, and polyphenol interaction with other food matrixes based on high or low water contents [24,25]. Multiple studies have focused on testing total phenolic content and antioxidant activity in *M. flexuosa*, but a comparison of the influence in dehydration states in pulp or seeds and its connection to antioxidant activity is still lacking in information and research [26,27,28,29]. The contradicting results show that some studies highlight decreasing TPC and higher antioxidant activity, while others report the opposite situations tested in a different food matrix, such as grapes, onions, rice, and other vegetables [30,31,32,33]. The goal of the present study was to determine a comparative approach to polyphenols from *M. flexuosa*, with high and low water content (fresh and de-hydrated samples), under active interconversion, and their relation to effective antioxidant activity.

## 2. Results and Discussion

### 2.1. Food Matrix Dehydration and Polyphenol Extraction

**Figure 2:**
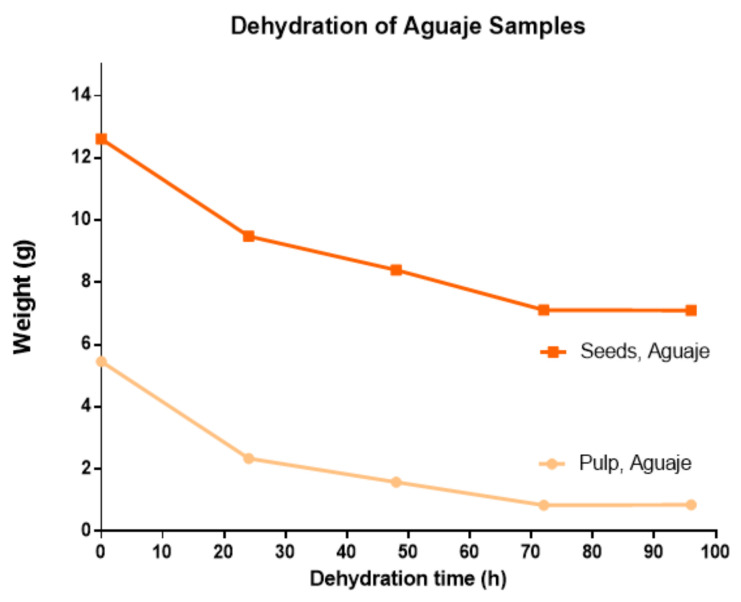
Dehydration curve for *M. flexuosa*, pulp and seeds at a constant 50 °C from 0 to 96 h.

Samples of *M. flexuosa* pulp and seeds were treated by extraction and a controlled dehydration process at four different times (24, 48, 72, and 96 h). The biggest weight difference was recorded for the first 24 h of dehydration. The water content in pulp and seeds was approximately 85% and 44%, respectively. Dehydrated weights were stable at 96 h at 50 °C. Considering the high difference in water content for pulp and seeds, the dehydration pattern (Figure 2) yielded similar values.

**Figure 3:**
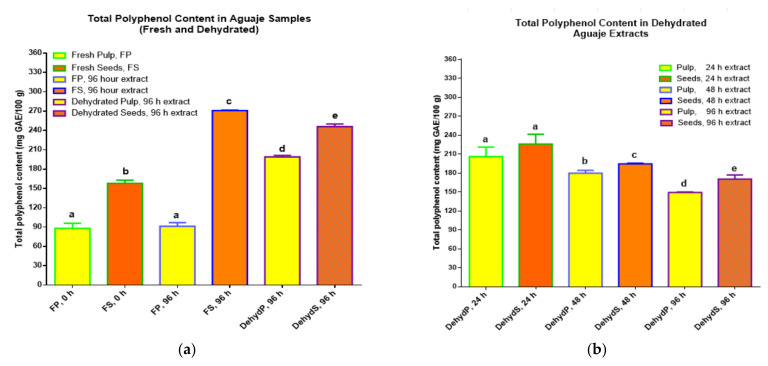
TPC (mg of gallic acid equivalents per 100 g) of fresh and dehydrated pulp and seeds of (**a**) *M. flexuosa* (aguaje) fresh and dehydrated pulp and seeds for the initial experimental assay and (**b**) TPC in *M. flexuosa* dehydrated pulp and seeds in a second assay. Data are means (±standard deviation), and lower-case letters represent significant differences, for total polyphenol content in mg GAE/100 g of initial sample, based on ANOVA followed by Tukey test (*p* < 0.05).

Polyphenols from fresh pulp and seeds were extracted in ethanol (80% *w*/*v*) and more polyphenol extractions were done with seeds and pulp at three dehydration intervals (24, 48, and 96 h). The comparative extraction looked for differences in polyphenol content, based on conflicting studies that reported higher phenolics and antioxidant activity in fresh fruit, seeds, or peels [34,35]. Figure 3 shows the comparative values for total polyphenol content from pulp and seeds in fresh or dehydrated form.

### 2.2. Total Polyphenol Content (TPC)

Polyphenols that were initially extracted from the fresh pulp of aguaje at 0 h of dehydration and the samples of fresh pulp that continued with the extraction process for another 96 h, in the dark without stirring, registered the lowest TPC with statistical significance, with respect to the phenolic content in fresh and dehydrated (96 h) seeds, registering approximately 90 mg GAE/100 g. The highest TPC was detected in fresh and dehydrated aguaje seeds (dehydration at 96 h) yielding more than 270 mg GAE/100 g in fresh seeds, as shown in Figure 3a that represents the results for the first experimental assays. The best aqueous conditions for polyphenol detection and reactions (fresh pulp matrix) did not favor higher TPC. These results suggest, coinciding with other researchers, that higher phenolic presence is more connected to the food matrix and the solvent affinity due to polyphenol polarity [35]. Nevertheless, in comparison with other fruits, we registered a high TPC; other studies reported *M. flexuosa*, with TPC values of 435.08 ± 6.97 and 362.90 ± 7.98 mg GAE/100 g of the whole pulp in fresh form [29,36]. The results here differed with studies where the TPC in fresh fruit, after food processing, was lowered or lost due to temperature or processing changes [31,32].

For the second experimental assay, concerning the TPC results for dehydrated *M. flexuosa* pulp and seeds, as shown in Figure 3b, a regular decreasing TPC pattern from 24 to 96 h of dehydration was registered. TPC values at the three different dehydration points were higher than the TPC in fresh pulp and seeds; the highest TPC was quantified at 24 h of dehydrating pulp and seeds at a constant 50 °C. The results at 48 and 96 h yielded slightly lower TPC values with statistical significance. The lowest phenolic content was measured in dehydrated pulp at 96 h (149.28 ± 0.81 mg GAE/100 g). This decreasing trend in phenolic concentration is consistent with research done with the purpose of evaluating TPC values in fruits processed with long effective storage time for exportation purposes [31].

### 2.3. Antioxidant Activity, DPPH (2,2-Diphenyl-1-picrylhydrazyl) Radical Scavenging Assays

**Figure 4:**
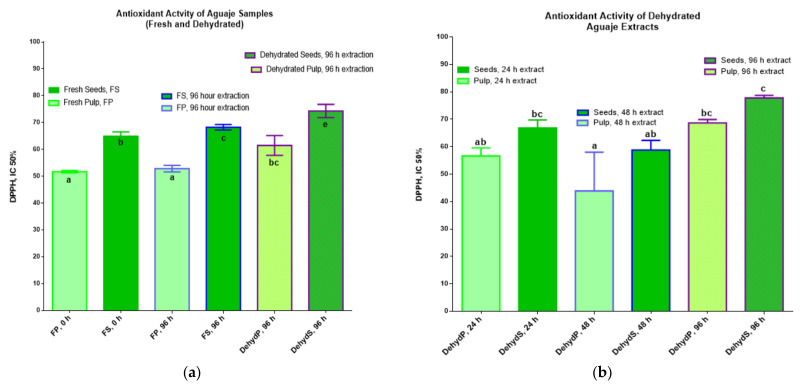
Antioxidant activity (mg AAE/100 g FP) of extracts in two different experimental assays. (**a**) fresh and dehydrated *M. flexuosa*) and (**b**) dehydrated pulp and seeds at different times. Data are means (±standard deviation) and lower-case letters represent significant differences, for antioxidant activity of aguaje extracts according to DPPH (IC 50%), based on ANOVA followed by a Tukey test (*p* < 0.05).

The results for the antioxidant action of phenolic compounds, extracted in ethanol 80%, in a fresh and dehydrated matrix at different times for *M. flexuosa* (aguaje) samples are shown in Figure 4, showing the comparative results for two different experimental assays. The total inhibitory concentration, IC 50%, that yielded the best output was due to phenolics in dehydrated seeds at 96 h (78.28 ± 0.67 mg AAE/100 g of dehydrated sample). All DPPH results were higher in the extractions from fresh or dehydrated seeds. In both assays, Figure 4a,b, the antioxidant activity registered an increasing tendency. The best DPPH results, with statistical significance, were detected in *M. flexuosa* pulp and seeds dehydrated for 96 h.

**Figure 5:**
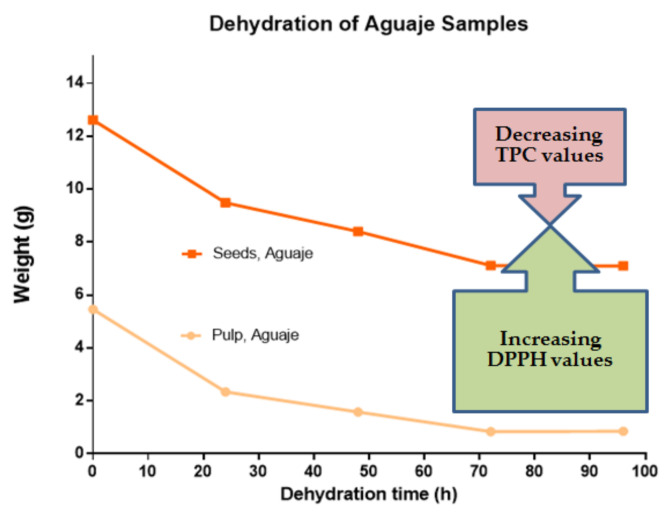
TPC (mg of gallic acid equivalents) and DPPH (mg ascorbic acid equivalents) in *M. flexuosa* (aguaje) pulp and seeds with respect to a constant dehydration pattern from 0 to 96 h. The stable flattening dehydration pattern is connected to lower TPC values and higher redox activity. From 72 to 96 h, the weight differences were stable, and statistical significance was registered at different points in the dehydration process.

These results for antioxidant activity coincide with the assays, in the same fruit, where other parts of the plant were evaluated [26,37]. The dehydration approaches in the present work led to improved DPPH values, with respect to the previously cited studies. The results are comparable and coincide with studies applying more complex dehydration processes [38,39]. Figure 5 represents a summary display of the dehydration process with respect to TPC and antioxidant activity seen in *M. flexuosa* pulp and seed extracts, as a comparative base for the various other studies.

Studies evaluating the biological activity of polyphenols in red raspberries showed that dehydration led to a lowering polyphenol content and less antioxidant activity, while the rate of this reduction was connected to the dehydration method, and this showed that a high dehydration temperature was linked to polyphenol content loss [40,41]. Increasing temperature promotes higher solubility and diffusion coefficient of polyphenolic compounds into the extracting solvents, and higher temperatures also enhance the penetration of solvents into the cell matrix; hence, increasing the TPC of the extracts [41].

Furthermore, the results confirmed a positive correlation between the total polyphenol content and the antioxidant activity for the aglycone compounds undergoing the drying process (74.7%). These lowering TPC values may be due to oxidative and thermal degradation of the phenolic compounds [42,43]. The major phenolic content drop, during fruit processing at high temperatures in enzymatic reactions, could be related to the action of oxidative enzymes such as polyphenol oxidases (PODs) and polyphenol peroxidases (PPOs) [43,44]. PODs can enhance the degradation of phenols when coexisting with PPOs; both the PPO and POD enzymatic activities play a key role in determining the phenolic profile of olive oil. In contrast, low temperatures during the dehydration process decrease the oxidation of volatile compounds [45].

When Cabernet Sauvignon and Merlot grapes were dried using a constant low temperature (7 °C) for several weeks, there was an increase in TPC and antioxidant activity [46]. This could be due to the effect of the concentration of the phenolic compounds as a result of water loss caused by the dehydration, even if the constant temperature (40 °C) was higher [47]. The results in these two previous studies partially coincide with the findings in the present work, with respect to the higher antioxidant activity of polyphenols in dehydrated aguaje fruit samples, but differ with respect to the decreasing phenolic content during d the dehydration process.

In a study evaluating the redox activity of phenolics from goldenberry, the levels of TPC and antioxidant activity (determined with the ferric reducing antioxidant power FRAP method) increased in dehydrated samples [20]. Furthermore, the highest TPC was registered in samples that were dried at 90 °C, coinciding with the results in this study, where samples were dehydrated at lower temperature (50 °C). The interconversion of phenolic compounds at high temperatures might be caused by the availability of phenolic precursor molecules through the non-enzymatic rearrangement between phenolic molecules [48]. This higher phenolic content may originate from the disruption of cell walls during processing or the breakdown of insoluble phenolic compounds. Therefore, this could lead to a better extractability for these particular types of phenolic compounds [49].

These comparative studies suggest that the antioxidant activity could be due to the combined reactions of total phenolics rather that certain individual components or the action of polyphenols as a whole group. The results in this study, using fresh and dehydrated aguaje seeds and pulp, coincide with the findings of Gupta et al. (2021), testing different parts of pomelo fruit (*Citrus grandis* (L.) Osbeck) in similar experiments for antioxidant activity. They found that TPC was highest in the membrane of the fruit, and DPPH registered the highest activity in the pomelo juice [50]. Considering the total polyphenols in the present study, the improvement in TPC could be a result of the destruction of the covering structure and the release of more phenolic compounds, facilitating and increasing the extraction yield [48]. Moreover, the dehydration process could induce metabolic pathways that can generate, and increase the number of, precursors for different categories of phenolic compounds [49].

## 3. Materials and Methods

### 3.1. Reagents and Chemicals

Ethanol, sodium carbonate, and Folin–Ciocalteu reagent were purchased from PanReac AppliChem, ITW Reagents, (Darmstadt, Germany), and methanol, from Sigma Aldrich (St. Louis, MO, USA). Ascorbic acid (Sebion) and DPPH (2,2-Diphenyl-1-picrylhydrazyl, Sigma-Aldrich) were purchased from Merck KGaA (Darmstadt, Germany).

### 3.2. Plant Material, Sample Preparation, and Polyphenol Extractions

*M. flexuosa* pulp with seeds was obtained in the Colombian city of Leticia. All samples were refrigerated before laboratory analyses. The extraction of total polyphenol compounds was performed following a previous method from this research group [2,51]. Different amounts of fresh and dehydrated pulp and seeds of *M. flexuosa* were placed in ethanol (80% *w/v*) at proportions of 1:5 per volume, stirred for 15 min at 500 rpm, homogenized, and stored at room temperature, in the dark, for 24 h without stirring. Two different extractions times (—*al fresco*—0 h and 96 h of extraction) for the first experimental assay, as an explorative comparison, and three different dehydration times (24, 48, and 96 h) at a constant of 50 °C, including only samples in dry state as a second assay, were considered in this experimental process. The extracts were centrifuged for 10 min at 3500 rpm, and the supernatant was recovered for polyphenol quantitation and antioxidant activity evaluation.

### 3.3. Total Polyphenol Content (TPC)

The total polyphenol content in *M. flexuosa* pulp and seeds (fresh and dehydrated matrix) was quantified following the Folin–Ciocalteu (F–C) assay [51]. Samples of 1 mL of each extract were mixed with 1 mL F–C reagent (10% *w*/*v*), allowed to react for 2 min, and mixed with 2 mL sodium carbonate, Na_2_CO_3_, (3.5% *w*/*v*). Reactants were kept in the dark at room temperature for 90 min. All runs were performed in triplicate. Absorbance was read at 655 nm in a UV-Vis spectrophotometer (Mecasys Optizen POP, Daejeon, Korea). All data were calculated based on a gallic acid standard calibration curve, with a range of 0–4.0 mg/L and r^2^ of 0.9982). TPC is expressed as milligrams gallic acid equivalent (GAE) per 100 g of fresh or dehydrated sample (mg GAE/100 g).

### 3.4. DPPH Assay for Radical Scavenging Antioxidant Activity

The DPPH radical scavenging test is one of the most useful techniques to evaluate the antioxidant activity in polyphenols extracted from natural products. The DPPH compound is a stable free radical in methanol. The DPPH assay was performed following previous work from this research group [2]. Volumes of 1900 μL of DPPH (100 μM) prepared in pure methanol were mixed with 100 μL of each diluted (1:5) extract and left to react in the dark at room temperature for 30 min. The antioxidant activity from phenolics, in fresh and dehydrated pulp and seeds, of *M. flexuosa* was measured via spectrophotometry at 517 nm, comparing against a methanol blank. A positive control of ascorbic acid based on a calibration curve, and in triplicates for each colorimetric reaction, was applied in this methodology. The control curve was prepared with concentrations of comparable reference ascorbic acid (Merck KGaA, Darmstadt, Germany) in a concentration range from 50 to 600 μg/mL (r^2^ of 0.9945). All dilutions followed the same DPPH reaction conditions for the antioxidant activity evaluated in fruit extracts. The slope taken from the calibration curve served as the calculation of the inhibition concentration (IC 50%), when 50% of the antioxidant component was reduced. The results for IC 50% were determined based on the equation:

% scavenging DPPH free radical = (ABS_Control_ − ABS_Extracts_/ABS_Control_) × 100%

The antioxidant activity of *M. flexuosa* extracted phenolics is expressed as mg of ascorbic acid equivalents per 100 g of fresh or dehydrated pulp or seeds (mg AAE/100 g).

### 3.5. Statistical Analysis

All analyses were carried out in triplicate, and TPC and DPPH values are expressed as mean ± standard deviation (SD). Means were tested for normality and homogeneity. Data were analyzed based on a ANOVA test followed by Tukey test (*p* < 0.05) with the IBM SPSS Statistics software version 20.0 (IBM Corp., Armonk, NY, USA).

## 4. Conclusions

The total polyphenol content and antioxidant activity of *M. flexuosa* pulp and seeds, in fresh and dehydrated form, were tested in this work. The water content in a food matrix allows for a specific polyphenol oriented chemical reaction, yielding better results in some cases where the water content is higher. A controlled dehydration process was considered in this experimental approach, with the purpose of evaluating the polyphenol availability and antioxidant action. TPC values were not directly proportional to antioxidant activity, suggesting that the polyphenol reactions for radical scavenging in pulp and seeds of *M. flexuosa* do not depend directly in the specific quantity of phenolic compounds, but rather on the specific chemical structure or on its re-accommodation or interconversion. More studies based on the specific polyphenol/flavonoid content and the presence of other antioxidants, such as C vitamin, in *M. flexuosa* could lead to understanding more of the specific antioxidant activity of this fruit with multiple processes of it edible parts.

## Data Availability

The data presented in this study are available on request from the corresponding author.

## Sample Availability

Samples of the compounds are not available from the authors.
